# A Pest or Otherwise? Encounter of *Oryctes rhinoceros* (Coleoptera: Scarabaeidae) with Persistent Organic Pollutants

**DOI:** 10.3390/insects12090818

**Published:** 2021-09-12

**Authors:** Meng-Wei Shen, Hung-Chuan Chen, Shyi-Tien Chen

**Affiliations:** 1 Ph.D. Program in Engineering Science and Technology, College of Engineering, National Kaohsiung University of Science and Technology, Kaohsiung City 82445, Taiwan; i108109112@nkust.edu.tw; 2 Department of Safety, Health and Environmental Engineering, National Kaohsiung University of Science and Technology, Kaohsiung City 82445, Taiwan; u0113802@nkfust.edu.tw

**Keywords:** persistent organic pollutants (POPs), pentachlorophenol (PCP), polycyclic aromatic hydrocarbons (PAHs), dieldrin (DLN), *Oryctes rhinoceros* larvae, POP mass flow

## Abstract

**Simple Summary:**

A native, widely spread beetle, *Oryctes rhinoceros*, in Southeast Asia may clean up some of the persistent organic pollutants (POPs) for us if guarded in a controlled manner. Some xenobiotics persisting in our environment may cause harmful effects to the living creatures within their food web via a so-called “bioaccumulation effect”. The encounter of wild creatures with the POPs appears inevitable. Luckily, this study revealed that the proper breeding of the commonly seen beetle could degrade more than 95% of some studied POPs simply by ingestion. The beetle larvae tolerated different POPs at various extents, yet through an acclimation operation, the beetle’s mortality rate could be greatly reduced. Even though *O. rhinoceros* is considered a pest for some valuable corps, its removal of POPs in a natural, efficient and passive (i.e., fewer energy inputs) manner makes this alternative promising and deserving of further explorations.

**Abstract:**

The potential use of invertebrates as bioreactors to treat environmental pollutants is promising and of great interest. Three types of the persistent organic pollutants (POPs), namely pentachlorophenol (PCP), PAHs (naphthalene and phenanthrene) and dieldrin (DLN), were spiked in soil and treated by using *Oryctes rhinoceros* larvae, a known pest of coconut trees in southeast Asia, and also the indicators of POP toxicity and the fate and degradability of the ingested POPs were assessed. The larvae were tested at various levels of the POPs and went through an acclimation process. Without acclimation, the tolerance limits of the larvae toward PCP, PAHs and DLN were 200, 100 and 0.1 mg/kg-soil, respectively, yet with acclimation, the tolerance levels increased to 800, 400 and 0.5 mg/kg-soil, respectively. Biodegradation rates of all the tested POPs were >90% by week 2, with <5% and nearly 0% remaining in the feces and body of the larvae, respectively. The results suggest that the use of the beetle larvae in soil POP decontamination is doable.

## 1. Introduction

Long-term manufacturing and use of xenobiotic chemicals have resulted in the gradual accumulation of persistent organic pollutants (POPs) globally [1,2,3,4,5]. Ingestion of POPs by an organism evokes bioaccumulation [6,7,8,9], which generally results in more-or-less harmful effects to numerous organisms [10,11,12], especially to the ubiquitous beetle larvae. Due to their toxicity and retardation to degrade, several commonly encountered POPs, including pentachlorophenol (PCP), two polycyclic aromatic hydrocarbons (PAHs), i.e., naphthalene and phenanthrene, and dieldrin (DLN), were studied, and their adversary effects on the larvae of a regional beetle species, *Oryctes rhinoceros,* were assessed. Furthermore, the persistent existence of POPs poses a bigger hazard to the environment. To date, many remedial methods have been employed to resolve soil POP problems through chemical or biological treatments [13,14,15,16]; however, successful remedial cases in real field applications were minimal.

*Oryctes rhinoceros* is a well-known pest of coconut trees and also one of the most dominant beetle species in Southeast Asia. Its larval stage goes through three instars, and each takes roughly 2, 4 and 16 weeks, respectively, to complete. Due to its pest nature, insecticidal compounds, including 22-hydroxyhopane from *Adiantum latifolium*, have been used in its population control [17]. On the other hand, two potential roles regarding *O. rhinoceros* have been reported: one was as the nutritional source of protein [18] and the other as the bioreactor for pollutant’s decontamination [19]. In either case, precautions of stopping the larva from escaping need to be known. For more information regarding the life cycle of *O. rhinoceros*, beneficial uses of its larvae and their potential applications and problems, refer to Shen et al. [19].

When the POPs and the *O. rhinoceros* encounter each other, two major concerns of the POPs to beetle larvae were brought about immediately: one is the toxicity of the POPs and the other the beetle’s adaption to the POP-contaminated environment. Based on the life cycle of the larvae, a long, nearly six months of the larval period gives them more time to adapt to toxin consumption and also allows them to ingest/degrade quite a great deal of the pollutants. The extents of ingestion and/or digestion of the POPs by the larvae have not yet been studied and were worthy of further investigation. To assess the adversary effects of the POPs, a mimicked larval breeding environment was constructed. Several designated incubators were set up with fixed amounts of substrate and various types of POP-contaminated soils. Five indicators: the larval death rate, growth rate, cumulative feces production, substrate conversion ratio and percent of the POP degradation, were monitored weekly over the entire testing period. By doing so, the toxicity, the larval adaption and the degradability of the POPs were to be accessed. Mass flow of each studied POP was also traced to confirm the biodegradability of the tested POP.

Upon the completion of this study, the extent of the POP’s impact, the ability of larval adaptation to the POPs and the degradability of the POPs by the beetle larvae were to be fully understood. This information is deemed as important data on the management of the POP-contaminated sites, on the evaluation of the larval survival and the further applications of the POP-contaminated soil cleanups. Although the use of rhinoceros beetle larvae as bioreactors in treating environmentally persistent pollutants has not yet been demonstrated. Use biological treatment alternatives are commonly regarded as environmentally friendly and cost-effective alternatives. The use of large insects as bioreactors on pollutant removal via proper site management operations looks promising.

## 2. Material and Methods

The tested larvae were captured from their wild habitats, mainly from decayed wood stumps, and bred using a commercially available larval substrate, namely the fermented product of dust wood, leaves and soil. For information regarding an overall larva’s handling process and life span, please refer to Shen et al. [19]. The spiked chemicals, namely PCP, PAHs and DLN, were purchased from Sigma-Aldrich, Inc. A 2 mm screen sieved, silt-clay soil separately spiked with PCP, 2 PAHs and DLN at concentrations of 5000, 70,000 and 200 mg/kg-soil, respectively, was mixed in a tumbler for 2 days before use. In PAH-spiked soil, 2 PAHs (i.e., NAP and PHE) were tested so that each PAH contributed half of the PAH content (i.e., 35,000 mg/kg) in soil. Hereinafter, the PAH content in soil is presented as the sum of the 2 PAHs. In each incubator setup, equal weights of the toxin-contaminated soil and larval substrate were mixed to obtain the required toxin levels. A list of the tested POPs is given in Table 1.

A 37.5 × 23 × 15 (length × width × height in centimeters) rectangular plastic box (J004, CHEN JUNG Plastic Inc., Taiwan) served as the larval incubator. Several small holes were drilled on 2 sides of the incubator for ventilation. At time zero, each incubator was filled with 1.5 kg of the soil and substrate mixture with the designated toxin content, which was maintained at ambient room temperature and 45% moisture content during the experimental period. In approximately 2 months, 2 runs were conducted in a series. Run 1, the toxicity test, was conducted for 4 weeks for various PCP, PAH, and DLN contents, which were topped at 200, 800 and 2 mg/kg, respectively. The samples of the soil/substrate mixture and feed were collected every week and the residual POP amount was quantified. Run 2, the acclimation test, was conducted for another 4 weeks to confirm the effect of acclimation on the beetle larvae. The larvae, which experienced the POPs and survived in Run 1, were considered as acclimated ones. The mortality rates of the acclimated and unacclimated larvae were examined, and the weekly residual levels and fate of each POP during the experimental period were determined.

For the toxicity test (i.e., Run 1), 14 incubators were set up. Each incubator was filled with a 1.5-kg mixture of soil and substrate at a 1:1 ratio and 20 larvae. Among these 14 incubators, 2 were free of toxins and served as blanks, 3 contained PCP levels of 50, 100 and 200 mg/kg-soil, 5 contained PAH levels of 50, 100, 200, 400 and 800 mg/kg-soil and 4 contained DLN levels of 0.1, 0.5, 1.0 and 2.0 mg/kg-soil. In the acclimation and POP degradation test (i.e., Run 2), 14 incubators were set up, among which 2 were free of toxins and served as blanks, 4 contained PCP levels of 200 and 800 mg/kg-soil, 4 contained PAH levels of 100 and 400 mg/kg-soil and 4 contained DLN levels of 0.1 and 0.5 mg/kg-soil. In other words, two incubators each received 15 either acclimated or unacclimated larvae in each POP level were arranged so that whether the toxin-exposed larvae survived exposure to a higher level of toxins could be tested. The acclimated larvae were the surviving larvae from the toxicity test that underwent exposure levels of 200, 50 and 0.1 mg/kg-soil of PCP, PAHs and DLN, respectively, for 4 weeks.

During each weekly sampling of the incubator operation, the incubated larvae were removed from the incubator and weighed, and the average weight of the larvae was determined to minimize disturbance. Subsequently, the larval feces (approximately 8–10 mm in size) was screened and separated from the substrate by using a 4-mm sieve. Finally, both the feces and substrate were oven-dried at 105 °C to determine their mass on a dry weight basis. By using the obtained data, the following 4 POP toxicity indicators were determined:

(1) $$\%\mathrm{Mortality}\;\mathrm{rate}\;\mathrm{of}\;\mathrm{larva},\;\%=\frac{\mathrm{total}\;\mathrm{number}\;\mathrm{of}\;\mathrm{death}}{\mathrm{number}\;\mathrm{of}\;\mathrm{larva}\;\mathrm{at}\;\mathrm{time}\;\mathrm{zero}}\times 100\%$$

(2) $$\mathrm{Weight}\;\mathrm{increase}\;\left(w.r.t\;\mathrm{initial}\;\mathrm{weight}\right),\;\mathrm{times}=\frac{\mathrm{weight}\;\mathrm{of}\;\mathrm{larva}\;\mathrm{at}\;\mathrm{sampling}\;\mathrm{time}}{\mathrm{initial}\;\mathrm{weight}\;\mathrm{of}\;\mathrm{larva}}\times 100\%$$

(3) $$\mathrm{Specific}\;\mathrm{feces}\;\mathrm{production},\;g/\mathrm{larva}=\frac{\mathrm{total}\;\mathrm{weight}\;\mathrm{of}\;\mathrm{feces}}{\mathrm{number}\;\mathrm{of}\;\mathrm{larva}\;\mathrm{survived}}\times 100\%$$

(4) $$\mathrm{Substrate}\;\mathrm{conversion}\;\mathrm{ratio},\;g/g=\frac{\left(\mathrm{larval}\;\mathrm{weight}\;\mathrm{at}\;\mathrm{sampling}\;\mathrm{time}-\mathrm{initial}\;\mathrm{larval}\;\mathrm{weight}\right)}{\mathrm{substrate}\;\mathrm{consumed}}$$

To determine the POP amount, an additional 2 g of the soil/substrate mixture or feces were randomly sampled from the collected sample through extraction using 10 mL of a mixture of acetone and hexane at a 1:1 ratio (by volume) and sonicated using a sonicator (Misonix 3000). Subsequently, the sample was centrifuged at 10,000 rpm using a centrifuge (X-22R, Beckman Coulter Inc., Indianapolis, IN 46268, USA), and the centrate was analyzed using gas chromatography–flame ionization detector (GC-FID, 14B SHIMADZU Corp., Tokyo, Japan) equipped with a PET-5 capillary column from Sigma-Aldrich, Inc. The oven temperature was initially 50 °C for 5 min, then increased to 300 °C at a rate of 10 °C/min and then maintained at 300 °C for another 15 min before program termination. A fixed amount of the internal standard 2,3,4-tribromophenol was added to the centrate to calibrate analytical errors that may have occurred during the GC-FID analysis. For further information on sample extraction, analysis, and quantification, refer to Chen et al. [20].

To determine the weekly removal rate of POPs, the net difference between POP intake and excretion was calculated, and the resulting value was divided by the total intake (Equation (5)). To trace the distribution of each POP during Run 2, total POP intake and excretion, the POP amount in larval bodies and POP amount in gut contents were determined for each larva to assess the percent distribution of POPs in each portion of the specimen. The percent degradation of POPs was calculated using Equation (6).

(5) $$\%\;\mathrm{POP}\;\mathrm{weekly}\;\mathrm{removal}\;\mathrm{rate},\;\%=\frac{\left(\mathrm{weekly}\;\mathrm{POP}\;\mathrm{mass}\;\mathrm{intake}-\mathrm{excretion}\right)}{\mathrm{weekly}\;\mathrm{POP}\;\mathrm{mass}\;\mathrm{intake}}\times 100\%$$

(6) % POP weekly degradation, %=100%−% POP in larval body including in guts−% POP excreted

For data quality assurance and quality control, all measurements were performed in triplicate and presented as averaged values. Percentage error (% error) of each measurement was calculated by dividing the standard deviation by the mean of the measurements, and a 5% limit was adopted to ensure data consistency. As for experimental duplications, at least one duplicated incubator was duplicated in Runs 1 and 2, and average values were calculated and reported. Yet, for the clarity of the plotted figures, the standard deviation of the data was not plotted.

## 3. Results and Discussion

**Figure 1:**
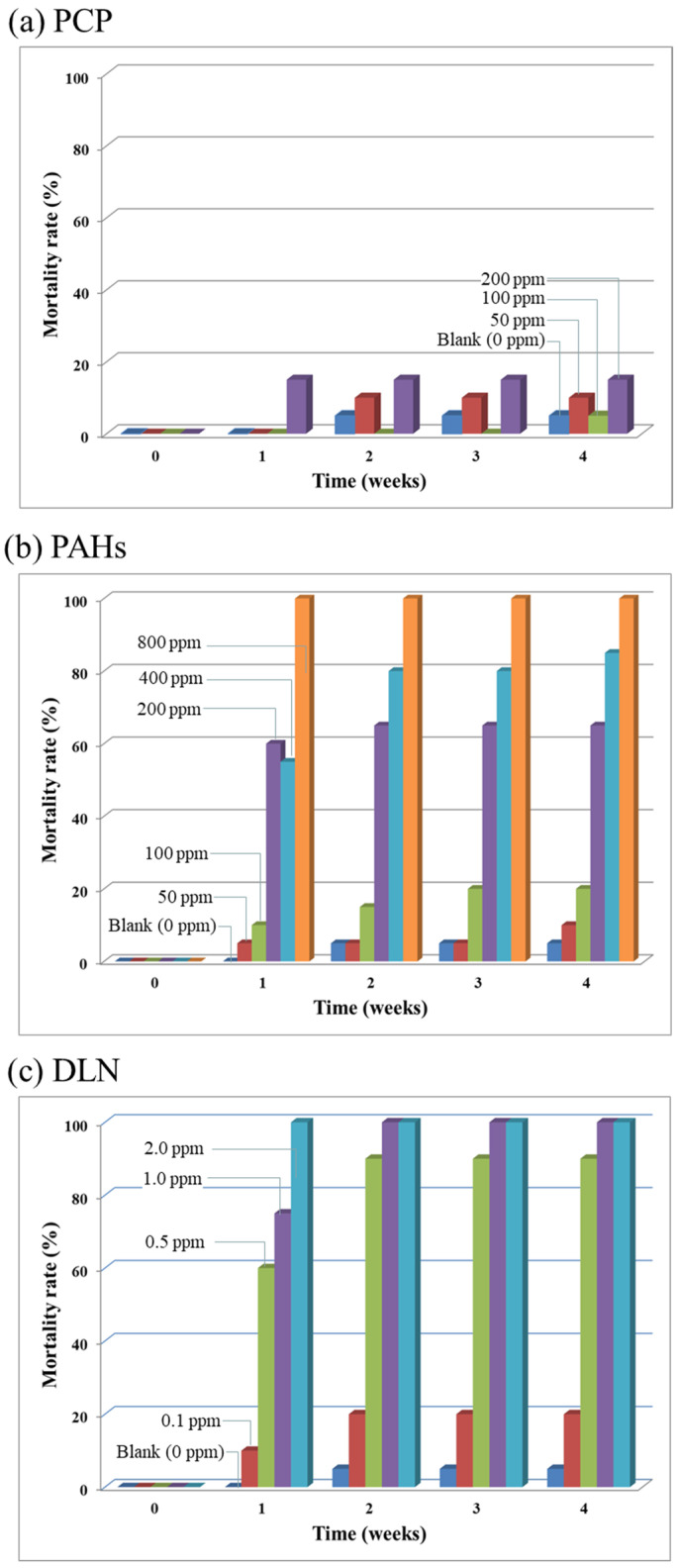
Mortality rates of beetle larvae in the presence of PCP, PAHs and DLN, as labeled in (**a**–**c**), respectively.

**Figure 2:**
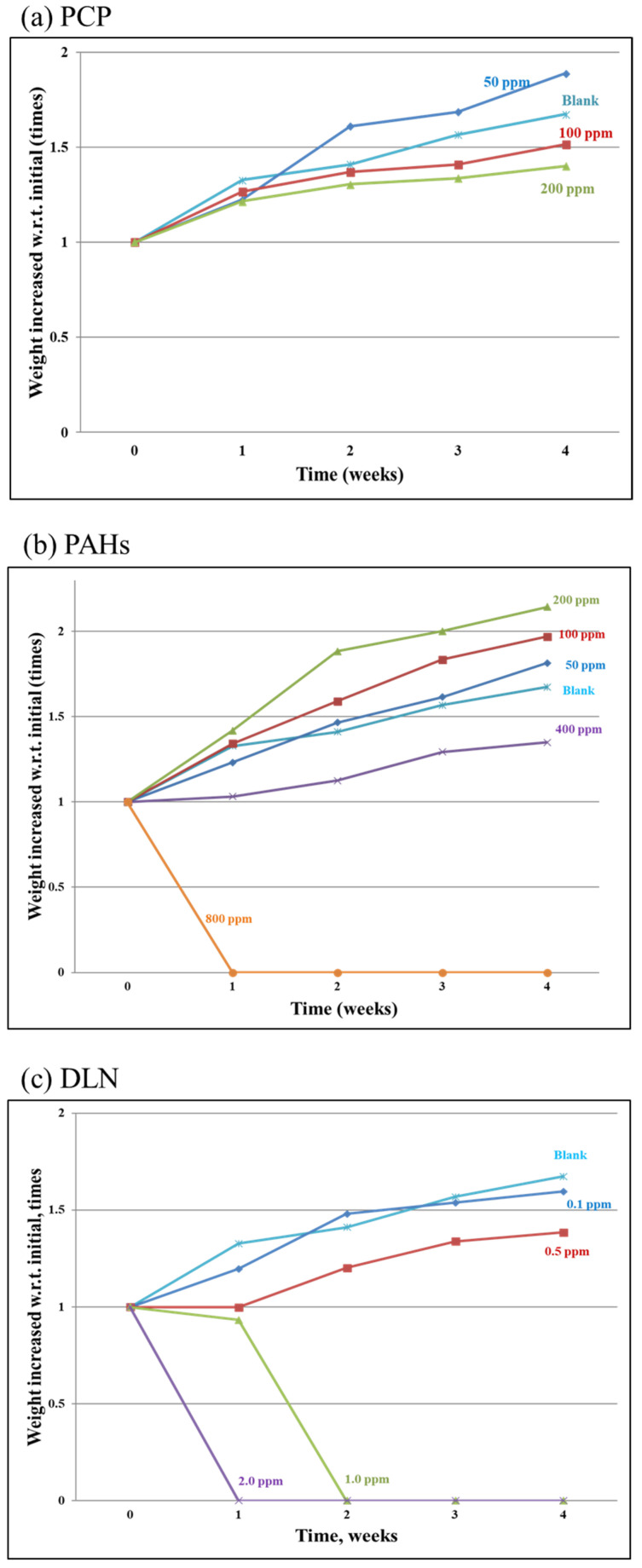
Increased larval weight with respect to its initial weight in the presence of the tested PCP, PAHs and DLN, as labeled in (**a**–**c**), respectively.

**Figure 3:**
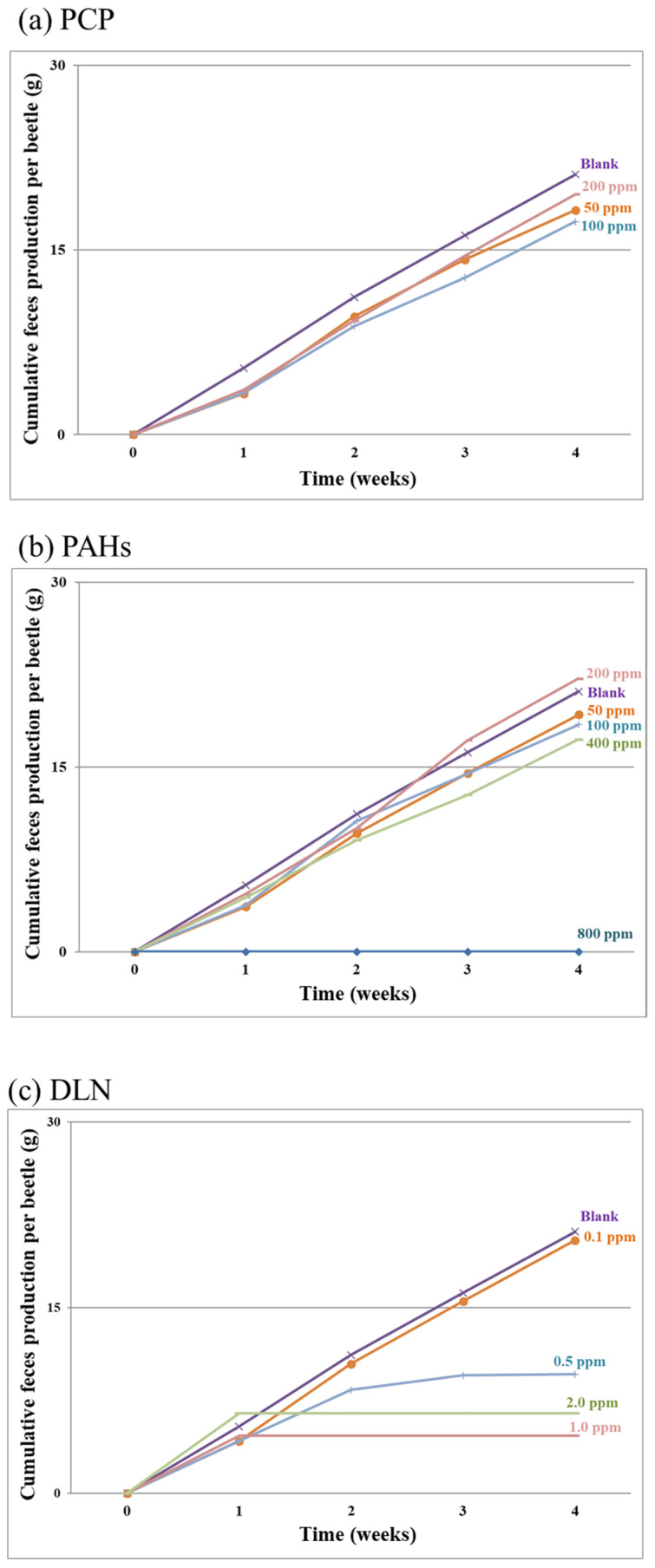
Cumulative feces production in the presence of the tested PCP, PAHs and DLN, as labeled in (**a**–**c**), respectively.

**Figure 4:**
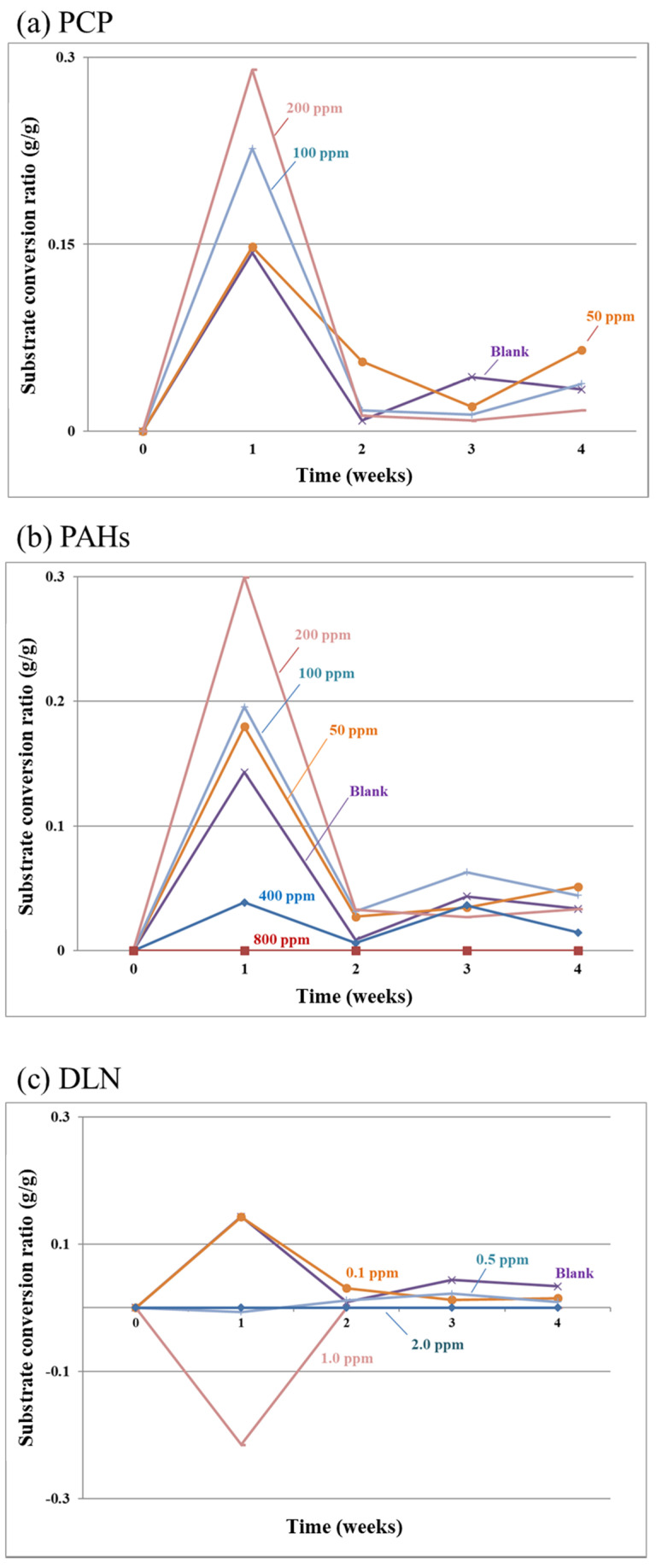
Substrate conversion ratio in the presence of the tested PCP, PAHs and DLN, as labeled in (**a**–**c**), respectively.

**Figure 5:**
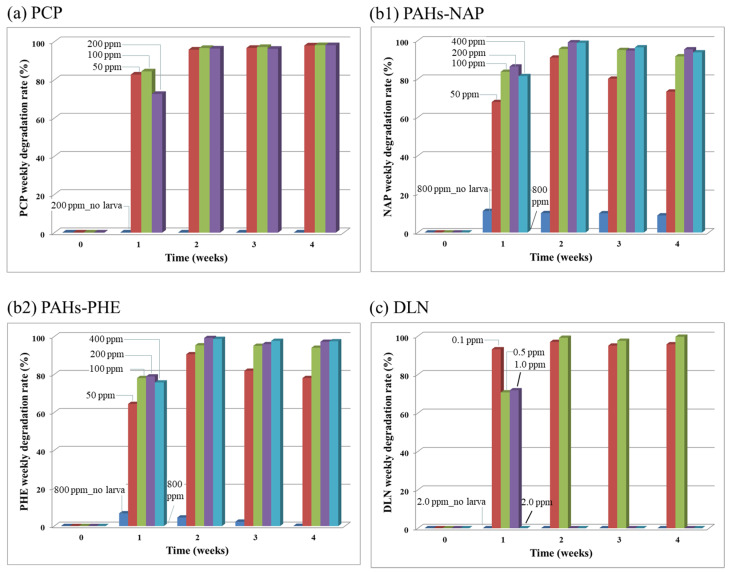
Weekly degradation rate of the tested PCP, PAHs-NAP and PHE and DLN, as labeled in (**a**), (**b1**) and (**b2**) and (**c**), respectively.

For the results of the toxicity test (Run 1), Figure 1, Figure 2, Figure 3, Figure 4 and Figure 5 illustrate the various growth effects of the tested beetle larvae due to the presence of each studied POP. Figure 1a–c depict the larval mortality rates caused by the presence of PCP, PAHs and DLN. Figure 1a reveals that a higher dose of PCPs corresponded to a higher larval mortality rate. However, up to 200-mg/kg of PCP, <20% larval mortality was observed, which was deemed to be a tolerable value in growing larvae. In the presence of PAHs (Figure 1b), the dose enhancement had a noticeable effect on mortality. When the PAH content equaled 200 mg/kg, >50% of the larvae died. When the PAH content reached 800 mg/kg, all the larvae died. Similar results were obtained for the addition of DLN runs, in which half of the larvae died when the DLN content was 0.5 mg/kg and all the larvae died when the DLN content was ≥1 mg/kg. Based on the lethal doses of the POPs, the DLN appeared more toxic than PAHs and PCP. Because of the large difference in toxicity dosage, the toxic mechanisms of each POP in larvae may merit further investigation.

Figure 2a–c illustrate an increase in larval weight concerning initial weights. In PCPs, all larvae that ingested >100 mg/kg PCPs demonstrated less body-weight gain over time. However, compared with the blank run, increases in larval body weight in the PCP-spiked incubators exhibited a similar trend and limited variations. In the incubators containing up to 400 mg/kg of PAHs, the larval body weight increased over time. However, when the spiked PAH content reached 800 mg/kg in the soil/substrate mixture, all the larvae died after 1 week of incubation; thus, the larval weight increase was zero (Figure 2b). Figure 2c illustrates the weight increase over time after DLN addition. The results demonstrate that the addition of two and one mg/kg DLN resulted in the death of all larvae in weeks 1 and 2, respectively. In general, higher POP content in the feed was associated with slower larval growth. However, in runs with a low PCP level or low PAH level (i.e., PCP or PAH incubators) the growth of larvae was faster than that in the blank run, which might be caused due to hormesis.

Figure 3a–c present the results of cumulative feces production per larva over time for the PCP-, PAH- and DLN-spiked incubators, respectively. Feces production exhibited a linear increase over time in most incubators, except for incubators with 100% larval death or a high larval mortality rate, such as in the 0.5 mg/kg DLN run. The data strongly suggest that the cumulative amount of feces might serve as a distinctive indicator of POP toxicity over the breeding period.

Figure 4a–c present the results of the substrate conversion ratio for the PCP-, PAH-, and DLN-consuming larvae, respectively. For the PCP consumption, most runs revealed a high substrate conversion ratio in week 1—some even higher than that in the blank run. This finding has two possible explanations: a small number of POPs appear to promote larval growth, and exposure to toxicity during the first week reduces substrate consumption and results in an increase in the conversion ratio. After week 1, the substrate conversion ratio exhibited trends similar to those of the blank run. The ratios were significantly lower in incubators with 100% larval death than in the blank incubators. For DLN contents of ≥1 mg/kg, the conversion ratios were nearly zero or even negative (weight loss). Overall, the four monitored indicators were considered sufficient for demonstrating the toxicity effects of POPs on larval growth and might be worth monitoring when breeding larvae in the presence of organo-toxins.

Figure 5a–c illustrates POP removal rates for the spiked PCP, PAHs and DLN, respectively. In the PCP-spiked incubators without the larvae (i.e., the Blank runs), the degradation rate of PCP at the 200 mg/kg level was nearly zero, and in incubators with the larvae, lower PCP degradation rates (approximately 80%) were observed for various PCP concentrations in week 1. During and after week 2, nearly 100% PCP removal was observed in all PCP-spiked incubators. The high-efficiency PCP degradation by larvae demonstrates their potential use for removing these types of toxins. Both the 2-ring NAP and 3-ring PHE exhibited similar degradation patterns. Because the PAH content of 800 mg/kg killed all the larvae in the incubator, no degradation was recorded. A rather contradictory phenomenon was observed: the incubators with low PAH content (i.e., 50 mg/kg) exhibited a low PAH degradation rate, which may be due to the no harm–no degradation scenario. Nearly 100% degradation of PAHs in incubators with a high PAH content (i.e., >100 mg/kg) indicated the potential use of these larvae as a remedial alternative. For the DLN incubators, the larvae removed DLN if the content was ≤0.5 mg/kg. However, at higher doses of ≥1 mg/kg, removal of DLN decreased, and the larvae eventually died in week 2. The level of DLN that larvae could withstand was considerably lower than those of PCP and PAH. The use of pesticides in agriculture planting might substantially affect the larvae’s survival in fields.

**Figure 6:**
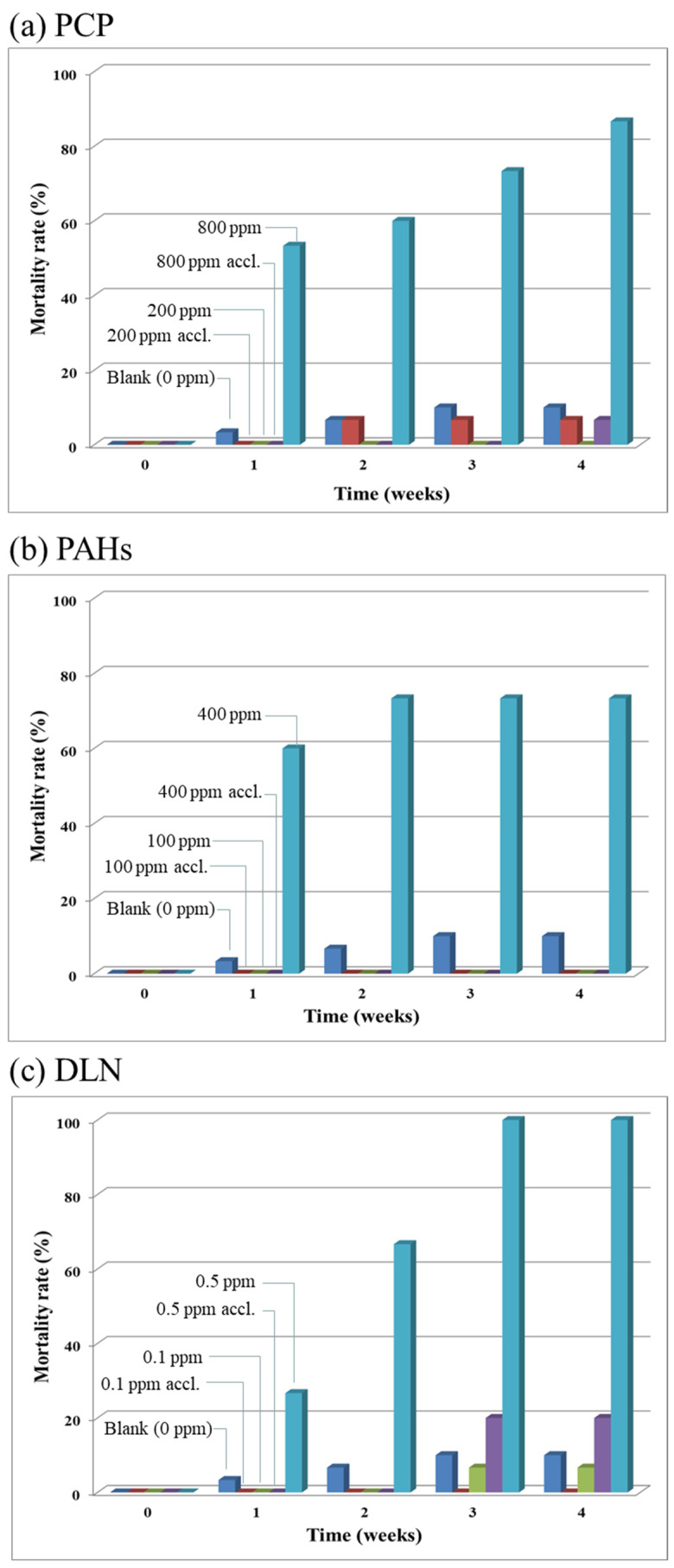
Larval mortality rates with or without acclimation to the tested PCP, PAHs and DLN, as labeled in (**a**–**c**), respectively.

**Figure 7:**
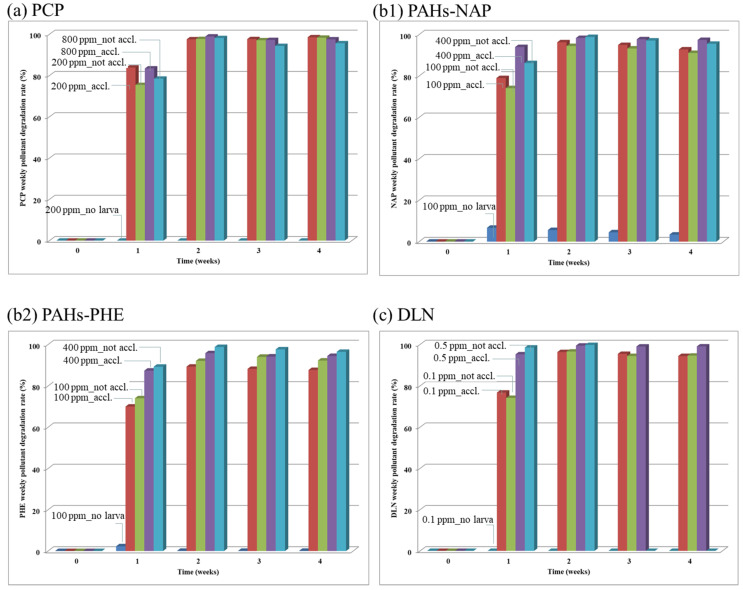
Weekly degradation rates of the tested PCP, PAHs-NAP and PHE and DLN, as labeled in (**a**), (**b1**) and (**b2**) and (**c**), respectively.

**Figure 8:**
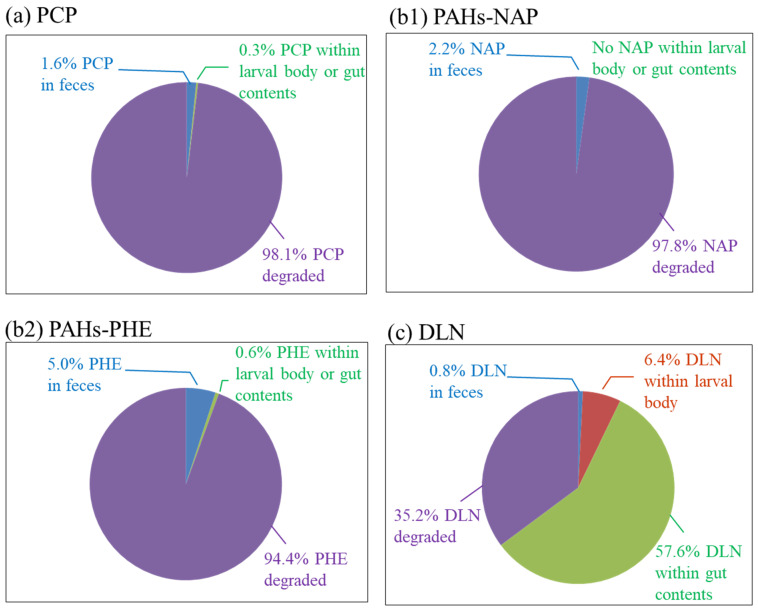
Mass flow of the tested PCP, PAHs and DLN, as labeled in (**a**)–(**c**), respectively, at the end of week 4.

As for the results of the acclimation and POP degradation test (Run 2), though the four POP toxicity indicators were also examined; however, due to their similar trends as concluded in the toxicity test, these data, except for the mortality rates, are not shown here. Thus, the larval mortality rates and weekly degradation rates of the spiked POPs are given in Figure 6 and Figure 7, respectively, and Figure 8 reveals the fate of each POP after its consumption by the larvae. The results in Figure 6a indicated that up to 80% of the larvae died at a high PCP concentration of 800 mg/kg in the soil/substrate mixture. However, larval death (i.e., ≤10%) was minimal when the larvae were exposed to PCP. For the 200 mg/kg level of PCP, the difference in mortality rates of the larvae with and without acclimation was small. The results revealed that as long as there were living larvae, PCP was being removed (Figure 7a). If the larvae could not degrade PCP, they died. Even some unacclimated larvae degraded PCP at a high level (i.e., 800 mg/kg); however, with acclimation, the mortality rate of the acclimated larvae was considerably low. Moreover, in PAH incubators, a high larval mortality rate was observed at a high PAH content of 400 mg/kg if the larvae were not acclimated and no mortality was observed if the larvae were acclimated. Both PAHs, that is, NAP and PHE (Figure 7(b1) and Figure 7(b2), respectively), exhibited >90% degradation if the larvae survived. A high larval mortality rate occurred if the larvae were not acclimated to PAHs beforehand. Similar results were obtained for DLN. Figure 6c shows high larval mortality for the unacclimated larvae at a DLN level of 0.5 mg/kg. More than 95% of DLN disappeared when the larvae survived. However, the larvae without acclimation removed a high percentage of DLN in weeks 1 and 2; however, because all unacclimated larvae died in week 3, the DLN removal rate decreased to zero (Figure 7d).

The fates of POPs were further studied by determining the amount of POP in larval feces, bodies and gut contents. In PCP incubators, 1.6% residual PCP was found in the feces and a total of 0.3% was observed in larval bodies and gut contents, which indicated that 98.1% of PCP was degraded through larval ingestion (Figure 8a). In PAH incubators, approximately 97.8% of the 2-ring NAP was degraded. A total of 2.2% of the NAP was remaining in larval feces, but no residual NAP was observed in larval bodies or gut contents. For the 3-ring PHE, approximately 94.4% was degraded, 5% remained in the feces and only approximately 0.6% remained inside larval bodies. Compared with PCP and PAH incubators, a large decrease in DLN degradation of 35.2% was observed through mass balance analysis. A large amount of DLN (approximately 57.6%) was present in gut contents, and approximately 6.4% and only 0.8% DLN remained in the larval bodies and feces, respectively. The results revealed that low amounts of DLN in larval feces gave a false DLN removal rate in the toxicity test. The high percentage of DLN in the larval bodies and gut contents indicated that the larvae accumulated DLN instead of degrading it after ingestion, which could be the main reason for the resulted larval death at low DLN concentrations.

Up to date, studies directly related to *Oryctes rhinoceros* encountering POPs are limited. Results of this study revealed that the impacts of POP dosages on the larval growth indicators looked obvious and indisputable. Once a higher and higher amount of POP was ingested, larvae’s mortality became the final resolution of such an encounter. However, based on the obtained results, the use of beetle larvae in environmental toxin removal appeared promising in three observations: (1) the larval survival rates and POP tolerance levels increased if larvae properly acclimated, which revealed the potential elimination of POPs in the fields through certain site management, (2) the four POP toxicity indicators might serve as operational monitoring items during the field remedial treatment, and finally (3) the complete degradation of the POPs makes the larval remediation a natural and environmentally friendly alternative.

The potential use of beetle larvae to clean up our environment sounds non-realistic. Yet, some characteristics of invertebrates, such as their long-term inhabitation in soil, capacity to chemically adapt, their high production rate and their global presence, make them excellent candidates in soil decontamination and this kind of decontamination might have happened without our notice. As a similarity, the earthworms have been widely studied for soil pollution treatment [21,22,23,24,25,26,27]. With a comparison to the studied larvae, the treatment of several milligrams of POPs per kilogram of soil using earthworms [28] has limited its application in a POP-contaminated site. Both methods (i.e., the beetle larvae and the earthworms) are considered passive treatment alternatives due to their minimum requirements in energy inputs. However, the beetle larvae could tolerate much higher POPs and appeared to be more applicable in dealing with highly POP-contaminated sites. In addition, Shen et al. [19] proposed the use of *Oryctes rhinoceros* as a bioreactor to treat total petroleum hydrocarbons (TPHs). Their results revealed that with acclimation, beetle larvae could stand high doses of TPHs and resulted in a higher TPH removal efficiency over time. This implied the potential uses of the beetle larvae as bioreactors in treating various pollutants.

Even the obtained results look encouraging, yet some precautions deserve further considerations. Firstly, *O. rhinoceros* is a pest in nature. Certain risks exist if used in soil decontamination. To fully adopt the larval bioreactors in pollutant decontamination, both human health and ecological risk assessments are of concern. For detailed risk assessment information, refer to the USEPA [29], and for a specific case study of assessing health risk characterization of some POPs, refer to Li [1]. From an engineering point of view, to lower the risks, an ex situ treatment can be done by isolating the POP-contaminated soil and the larvae in a close system to avoid the escaping of the beetle larvae and adults into the wild. Secondly, high larval mortality due to certain POPs such as DLN in this study might prohibit the employment of beetle larvae at work. Whether a longer acclimation time would help needs to be confirmed. At last, questions like the optimal means to breed the larvae on-site, the upper limits of a treatable POP level and the possible containment to limit the escape of larvae if the contaminated soil is treated in situ, etc. need to be answered. Thus, further studies are believed necessary before the proposed remedial operation becomes workable.

Two references were added in the last paragraph of the “Results and Discussion” section regarding the risk assessment issue. One was the USEPA risk assessment website (https://www.epa.gov/risk, accessed on 31 August 2021) that fully explains the contents of human health and/or ecological risk assessments, and the other by Li [1], who reported a case study on health risk characterization on persistent organic pollutant (POP) pesticides in residential soil.

## 4. Conclusions

Based on the aforementioned results and discussion, the following conclusions were drawn: (1) without exposure to POPs, the tolerance levels of larvae toward PCP, PAHs and DLN were 200, 100 and 0.1 mg/kg-soil, respectively. At or below such POP levels, the larvae exhibited <20% mortality and could be properly acclimated, (2) when the acclimated larvae encountered 800, 400 and 0.5 mg/kg-soil levels of PCP, PAHs and DLN, respectively, their mortality was minimal and >90% POPs were degraded by week 2. By contrast, at the same POP levels, most unacclimated larvae died. Appropriate acclimation of the larvae could stabilize the effects of real POP-contaminated field treatment, (3) the results of a fate analysis revealed that >98% and >95% of PCP and PAHs, respectively, were degraded through the larval intake, with a minimal POP amount remaining in larval feces and bodies, and (4) by contrast, only approximately 35% of DLN was degraded through larval ingestion. Nearly 58%, 6.4% and 0.8% of spiked DLN were accumulated in larval gut contents, bodies and feces, which indicated a large accumulation of DLN in the larvae.

Based on these results, the degradation of certain POPs in the field might have already happened all the time. Yet through the uncovered data, the impacts of POPs to beetle larvae were revealed, proper site management via the proposed indicators learned and the potential of better performance on POP-contaminated soil remediation proposed. More studies at this point might help the larvae already there to recover our environment in a safer and faster way.

## Figures and Tables

**Table 1:** Some properties and regulatory limits of the tested POPs, including PCP, NAP, PHE and DLN.

Properties/Limits	Pentachlorophenol (PCP)	Naphthalene (NAP)	Phenanthrene (PHE)	Dieldrin (DLN)
Chemical structure	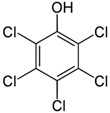 C_6_C_15_OH	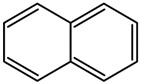 C_10_H_8_	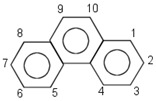 C_14_H_10_	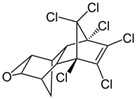 C_12_H_8_Cl_6_O
MT/BT (°C)	191/309–310	81/218	101/340	176–177/385
Solubility (mg/L)	14 (at 20 ℃)	32 (at 25 ℃)	1–1.3 (at 25 ℃)	0.195 (at 25 ℃)
Two-week LD_50_ (mg/kg)	27.3 (rat, oral); 96 (rat, skin)	1780 (rat, oral)	700 (rat, oral)	38.3 (rat, oral)
Soil regulatory limits in Taiwan (mg/kg)	200	Not applicable	Not applicable	0.04

MT/BT= Melting temperature/Boiling temperature.

## Data Availability

Data is contained within the article.
